# COVID-19 Anxiety—A Longitudinal Survey Study of Psychological and Situational Risks among Finnish Workers

**DOI:** 10.3390/ijerph18020794

**Published:** 2021-01-19

**Authors:** Iina Savolainen, Reetta Oksa, Nina Savela, Magdalena Celuch, Atte Oksanen

**Affiliations:** Faculty of Social Sciences, Tampere University, Kalevantie 5, 33100 Tampere, Finland; iina.savolainen@tuni.fi (I.S.); reetta.oksa@tuni.fi (R.O.); nina.savela@tuni.fi (N.S.); magdalena.celuch@tuni.fi (M.C.)

**Keywords:** COVID-19, mental health, anxiety, work, stress, personality, loneliness

## Abstract

*Background*: The COVID-19 crisis has changed the conditions of many all over the globe. One negative consequence of the ongoing pandemic is anxiety brought about by uncertainty and the COVID-19 disease. Increased anxiety is a potential risk factor for wellbeing at work. This study investigated psychological, situational, and socio-demographic predictors of COVID-19 anxiety using longitudinal data. *Methods*: A nationally representative sample of Finnish workers (*N* = 1308) was collected before and during the COVID-19 crisis. Eighty percent of the participants responded to the follow-up study (*N* = 1044). COVID-19 anxiety was measured with a modified Spielberger State–Trait Anxiety Inventory. Psychological and situational predictors included perceived loneliness, psychological distress, technostress, personality, social support received from the work community, and remote working. A number of socio-demographic factors were also investigated. *Results*: Perceived loneliness, psychological distress, technostress, and neuroticism were identified as robust psychological predictors of COVID-19 anxiety. Increase in psychological distress and technostress during the COVID-19 crisis predicted higher COVID-19 anxiety. A recent change in their field of work and decreased social support from work communities predicted COVID-19 anxiety. Women and young people experienced higher anxiety. *Conclusions*: Different factors explain workers’ COVID-19 anxiety. Increased anxiety can disrupt wellbeing at work, emphasizing the organizations’ role in maintaining an inclusive and caring work culture and providing technical and psychological support to workers during crisis.

## 1. Introduction

At the end of 2019, a new coronavirus SARS-CoV-2 was identified in Wuhan, China [1]. By March 2020, the novel coronavirus causing COVID-19 disease had caused a public health emergency and a global pandemic [2]. In response to the pandemic, numerous nations implemented stay-at-home orders and placed restrictions on events, services, and social gatherings to slow the spread of the disease [3,4]. The new guidelines with reinforced social distancing changed people’s daily routines and circumstances abruptly, which might have been challenging and psychologically demanding to many individuals. Having potential concerns about one’s own and loved ones’ health, being isolated and confined to one’s home, losing personal freedoms, and having no certainty in future plans are some added stressors brought by the pandemic [5]. These recent and concurrent changes may have also influenced people’s level of anxiety.

Under stressful or acute fear situations, anxiety is a normal response. This type of anxiety is described as a state that has a positive and motivating influence on adaptive behavior and coping [6]. A person may experience anxiety, for instance, before speaking publicly or taking a test. Once the situation has passed, state anxiety dissipates. However, anxiety can have different degrees of severity and involves a continuity concern [6]. As the current coronavirus pandemic continues, attention should be paid to the potential situational and psychological factors explaining COVID-19 anxiety. Emerging research has identified several factors influencing levels of psychological distress, fear, and COVID-19-related anxiety among members of the general public and older adults [7,8,9,10,11], but fewer studies have been conducted so far on COVID-19 anxiety and the related risk factors among working populations. This longitudinal research aims to fill this gap by investigating COVID-19 anxiety among Finnish workers.

### 1.1. Psychological Factors Explaining COVID-19 Anxiety

The COVID-19 pandemic has caused concerns about its influence on people’s mental health, creating a surge of research investigating potential factors explaining COVID-19-related distress and anxiety. One study surveying Chinese workers found that poor health status, drinking alcohol, and confirmed infections among one’s family and friends or in the community increased the risk for developing anxiety and depression symptoms during the first months of the pandemic [12]. Fernández and colleagues [13] found that female gender, young age, high neuroticism, and fear related to COVID-19 were associated with higher emotional suffering during quarantine, while higher income and being married protected adults from emotional distress. A similar study had analogous results, finding that females, younger individuals, loneliness related to the coronavirus situation, and having pre-existing chronic illnesses were related to higher levels of psychological distress during the pandemic [14]. Comparable results were obtained among Chinese front-line non-health workers, where young age, female gender and increased stress levels were, among others, identified as risk factors for developing depression symptoms [15]. The negative outcomes of loneliness on mental health during the pandemic were also observed in a sample of older adults, where perceived loneliness predicted COVID-19-related anxiety [7]. Furthermore, a longitudinal study on Chinese population found that trait loneliness had an impact on COVID-19-related anxiety [16].

The role of personality traits has been investigated in connection with coping with the pandemic and adherence to restrictions and social distancing guidelines e.g., [17,18,19], but fewer studies have examined the relationship between personality and COVID-19 anxiety. One study by López-Núñez and colleagues found that individuals low in neuroticism but high in conscientiousness, extraversion, and agreeableness had better mental health in terms of anxiety, depression, and life satisfaction during the COVID-19 outbreak in Spain [20]. Similarly, Nikčević and colleagues (2020) found neuroticism, as well as openness to experience, to be associated with higher levels of COVID-19 anxiety among the general population in the United States [21]. Agreeableness, extraversion, and conscientiousness had an opposite effect [21]. In a study conducted among young adults in India, higher neuroticism was connected to higher levels of death anxiety in the wake of the 2020 pandemic; however, this relationship was fully mediated by perceived stress [22].

These abovementioned findings support the prior literature indicating that individuals high in neuroticism respond more negatively to uncertainty [23]. The findings are further in line with studies on psychological flexibility and uncertainty tolerance, both of which have been linked to lower wellbeing and higher levels of anxiety during the COVID-19 pandemic [24,25,26,27]. One study found that lower emotional stability, including neuroticism, was associated with increased feelings of worry and stress during the pandemic [28]. In addition, intolerance of uncertainty was associated with higher levels of generalized anxiety and depression during the “lockdown” phase of the pandemic in the U.K. [29]. This finding was partially moderated by the tendency to choose maladaptive coping strategies [29].

Emerging evidence shows that different psychological, as well as personality and demographic factors, influence how individuals respond to the coronavirus pandemic. Consequently, it is imperative to extend our knowledge of the influence of the coronavirus crisis on working populations. Employees are an exceptional group of individuals to study, as they are simultaneously dealing with issues relating to work and the changes brought by the pandemic.

### 1.2. Wellbeing at Work

Mental wellbeing at work has gained a lot of attention in the media and research in recent years [30,31]. The COVID-19 pandemic has evidently put pressure on organizations to maintain a sense of community among workers and support the wellbeing of employees during remote work and unstable work situations. According to Eurofound (2020), during the COVID-19 crisis, 12% of EU workers have felt isolated and 25% have felt emotionally drained by their work [32]. However, the mental health of Finnish workers has not dramatically changed due to COVID-19 [33]. In fact, the mental wellbeing of EU workers improved overall during the April–July 2020 timeframe, Finland being among the five highest scoring countries on the WHO-5 mental wellbeing index [32]. However, many workers have experienced increased stress and lower wellbeing due to the impact of the coronavirus pandemic. Workers in the medical field have especially reported increased distress and levels of anxiety due to higher workloads and increased demands in work [34,35].

During the coronavirus crisis, working age people in Finland have been mostly concerned about infecting others or loved ones getting infected. In addition, they have worries about becoming sick with COVID-19, and whether the health care system is able to sustain the increased number of patients [33]. These concerns are very natural and can steer workers’ concentration away from their immediate work, hinder work performance, and create emotional burdens [36,37]. The concerns of workers also go beyond the immediate health-related worries of the global pandemic. For instance, perceived job insecurity was found to mediate the relationship between COVID-19-related fear and emotional exhaustion of front-line non-medical workers [38], which goes to show how economical concerns may negatively impact employees’ wellbeing during the current crisis. Another major cause of concern is the rapid and dramatic change in working methods. Knowledge workers in particular have increasingly worked remotely from home or in locations other than traditional workplaces [32]. Consequently, many organizations have taken into use new digital tools and platforms [39,40]. The fast digital leap has supported the completion of work and has maintained the connection to workplace and clients [37,41], but working through digital devices and having constant remote connectivity to a workplace can be increasingly exhausting experiences [39].

Maintaining social relations and feelings of belonging are fundamental elements for wellbeing [42] and can prevent the negative effects of experiencing loneliness at work [43,44]. Thus, social support from a work community has been an important resource for workers during the COVID-19 crisis. The role of social support has been studied in the workplace context during the pandemic to some extent [45]. For instance, perceived low support from a supervisor has been found to predict a range of negative mental consequences, including anxiety and depression among university faculty and staff [46]. Based on the Eurofound report (2020), 49% of Finnish workers reported receiving help and support from their managers and 60% from their colleagues during the crisis [32]. Encouraging and maintaining work engagement may be additional important factors, as low levels of work engagement and low sense of coherence have been associated with higher COVID-19-related distress among non-medical essential workers [47].

Technology has clearly assisted with receiving and maintaining these vital resources for work and wellbeing. However, not all workers are familiar and at ease with using technology, which can lead to technostress [48,49]. A recent study found that remote workers put in more work hours as they feel forced to work longer hours, and with higher workloads they experience more techno-stressors, such as invasion of technology into their home [50]. Back-to-back online meetings and virtual communication can be draining, and multitasking is common during online meetings as concentration can be difficult to sustain. This can lead to virtual meeting fatigue and lower wellbeing of workers [37,51]. Moreover, increased use of digital devices and spending increasing amounts of time online might lead to one-sided online interaction. As a result, individuals may become engaged in identity-driven social media bubbles. Social media identity bubbles refer to online activity which is characterized by social identification, homophily, and information bias [52]. Involvement in such social media identity bubbles reduces the likelihood of seeing ideologically diverse information [53].

### 1.3. Current Study

This longitudinal study set out to investigate what psychological, situational, and socio-demographic factors predict COVID-19 anxiety among Finnish workers. According to previous research, individuals have increased levels of anxiety due to the coronavirus pandemic, but more research is needed to understand the underlying risk factors as well as recognize potential protective factors of COVID-19 anxiety among workers. Experiencing anxiety related to the COVID-19 crisis might have a negative impact on workers’ overall wellbeing, but also disrupt their productivity and focus on work tasks. Consequently, the impact of COVID-19 anxiety among working populations could be felt on a larger societal level in terms of lost working hours and inefficiency [54].

Our study is theoretically grounded in research on wellbeing at work. Analyzing COVID-19 anxiety could help to understand psychological coping and protective factors under exceptional circumstances. This can have an impact on general theories of wellbeing at work. Research has thus far identified psychological factors such as fear and trait anxiety, as well as situational factors, such as living alone and having a pre-existing chronic illness, to predict COVID-19 anxiety among older adults, medical workers, and the general public [7,8,9,10,11]. In addition, women and younger individuals have been found more likely to experience COVID-19-related stress [13,14]. Therefore, we posed the following research question:

(1) How are psychological, situational, and socio-demographic predictors associated with COVID-19 anxiety among workers?

Given that the COVID-19 pandemic began in March 2020 and is still ongoing, we are interested in investigating changes in different psychological states and situational factors among workers, and their possible influence on COVID-19 anxiety. Thus, we formulated the second research question:

(2) Do changes in psychological and situational factors explain COVID-19 anxiety?

## 2. Method

### 2.1. Participants

Participants of the study took part in the longitudinal “Social Media at Work in Finland” survey, which was designed as a nationally representative survey of Finnish workers. In total, 1308 participants responded to the survey collected before the COVID-19 crisis between 16 September and 15 October 2019. Out of these participants, 79.82% (*n* = 1044) responded to the follow-up survey conducted between 15 September and 22 October 2020. The follow-up survey coincided with the second wave of the COVID-19 epidemic in Finland. In the first survey, the participants were 45.21% female, and between the ages of 18 and 66 (mean (M) = 45.02; standard deviation (SD) = 11.41). We detected no bias due to nonresponse and the sample does not include any major biases based on age and gender when compared with the official census figures of workers in Finland [53,55]. The sample of the study also matches the general Finnish working population and encompasses different occupational fields and educational levels. Further, the geographical distribution of the sample matches the Finnish working population [56].

### 2.2. Procedure

No ethical concerns were identified in the survey study design, as declared in December 2018 by the Academic Ethics Committee of Tampere region, Finland. The survey was designed by the research group and administered in Finnish. Participants were sampled, recruited, and administrated by a data-provider company Norstat and based on the target group of Finnish working population. Participation in the survey was voluntary and the goals of the research were explained to respondents.

### 2.3. Measures

#### 2.3.1. COVID-19 Anxiety

We measured COVID-19 anxiety with the six-item short-form of the state scale of the Spielberger State–Trait Anxiety Inventory STAI-6 [57]. The measure was adapted to the current COVID-19 situation by asking the respondents what kind of reactions the coronavirus crisis evokes in them (see Appendix A). They were then asked to evaluate how well the six statements described their state in the past seven days. The scale for each statement was from 1 (does not describe my state at all) to 7 (describes my state completely). Three of the statements were reversed for the analysis. The measure is not a diagnostic tool and has no set cut-offs. However, for the descriptive results we also report the proportion of participants having at least some anxiety by looking at the highest options from 5 to 7, on a scale from 1 to 7. See Table 1 for descriptive statistics of all measures. Internal consistency omega coefficients are also reported.

#### 2.3.2. Psychological Factors

Independent variables measuring psychological factors included perceived loneliness, psychological distress, technostress, work exhaustion, and personality. Loneliness was measured with the three-item loneliness scale adapted from the standard Revised UCLA Loneliness scale [58]. For psychological distress, we used the 12-item General Health Questionnaire [GHQ-12] with bimodal scoring (0-0-1-1) [59]. A social media technostress measure was adapted from Ragu-Nathan and colleagues’ technostress measure using six items on techno-overload and techno-invasion [60]. Work exhaustion was measured using a 5-item subscale of the 16-item Maslach and colleagues’ Burnout Inventory General Survey MBI-GS [61]. Finally, the personality traits of openness, conscientiousness, extroversion, agreeableness, and neuroticism were measured with the 15-item short measurement of the big five inventory BFI-S [62].

#### 2.3.3. Situational Factors

As situational independent variables, we used measures of social media information bubbles, social support from work community, remote work status, and living alone. A two-item information bias subscale was utilized from the Identity Bubble Reinforcement Scale IBR-S [52]. Social support received from work communities was measured with four questions included in the subscales of the Copenhagen Psychosocial Questionnaire (CPSQII). These questions focused on supportive working environments and support received from colleagues and supervisors [63]. In addition, we measured whether the participants work remotely and whether they live alone, both of which were used as dummy variables.

#### 2.3.4. Socio-Demographic Factors

In addition to gender and age, socio-demographic independent variables included the level of income, education, and occupational field. Age was used as a categorical variable with three categories: 18–29, 30–49, and 50–66 years old. Dummy variables were created for high income and education; value 1 indicates high income (5000 € or over/month) and having a university degree, respectively. Occupational field was measured with the Finnish version of the International Standard Industrial Classification of all economic activities ISIC [64], and were further classified into seven broader categories for analysis: manufacturing, service, business, communication and technology, public administration, education, health and welfare, and unknown or other.

#### 2.3.5. Changes over Time

Change in psychological distress, technostress, work exhaustion, social media information bubbles, social support at work, remote working, and employment status were measured between autumn 2019 and autumn 2020. In addition, the respondents were asked if they had changed occupational fields between the measurement times. Dummy variables were created based on the respondents indicating a change in any of the abovementioned measures. For example, those who had higher psychological distress during the COVID-19 crisis than before the crisis were categorized into an “increased psychological distress” group. We set those who did not show any change, or reported lower psychological distress, as reference groups.

### 2.4. Statistical Techniques

Multiple linear regression was based on ordinary least squares (OLS) regression. All regression models analyzed COVID-19 anxiety. All assumptions of OLS were met. Multicollinearity was not detected, and residuals were normally distributed. Heteroscedasticity of the residuals and outliers were also checked. Due to heteroscedasticity of the residuals in the full model of Table 2, we ran the models using Huber–White standard errors (i.e., robust standard errors). Due to the potential outliers, we also ran the analyses with robust regression, but this did not have an impact on the results.

We report unstandardized (B) and standardized (β) regression coefficients, standard errors (SE) of B and statistical significances (*p*) in the tables. In total, the study includes 1044 participants, but the number of participants vary from 883 to 1044 in different models. The full model includes 883 participants as personality information was only asked from 965 participants, and because some participants were no longer in the work life (e.g., became unemployed or retired) or did not respond to all the questions concerning wellbeing at work.

## 3. Results

Descriptive statistics of all measures are reported in Table 1. The analysis focused on COVID-19 anxiety which was a continuous variable with higher figures indicating higher anxiety. This scale had a mean of 18.91 (SD = 6.76) on a scale from 6 to 42. Half of the participants (50.86%) reported at least some COVID-19 anxiety when looking at the highest anxiety scores.

Regression analyses first investigated the associations adjusting for only age and gender (models 0 in Table 2). Out of the psychological factors, we found all variables to show statistically significant associations with COVID-19 anxiety, except openness to experience. Psychological distress (β = 0.36, *p* < 0.001), neuroticism (β = 0.31, *p* < 0.001), and perceived loneliness (β = 0.30, *p* < 0.001) had the strongest association with COVID-19 anxiety, but technostress and work exhaustion were also found to be associated with COVID-19 anxiety. Out of the situational factors, those participants who were in a social media information bubble (β = 0.08, *p* = 0.015) and worked remotely (β = 0.06, *p* = 0.043) reported higher COVID-19 anxiety. In addition, those who reported higher social support (β = −0.18, *p* < 0.001) reported lower COVID-19 anxiety. Women (β = 0.16, *p* < 0.001) and young people below the age of 30 (β = 0.09, *p* = 0.005) were more likely to report COVID-19 anxiety. Those with high income reported lower COVID-19 anxiety (β = −0.07, *p* = 0.029).

The full model reported in Table 2 showed that psychological distress (β = 0.17, *p* < 0.001), technostress (β = 0.17, *p* < 0.001), neuroticism (β = 0.17, *p* < 0.001), and perceived loneliness (β = 0.11, *p* = 0.005) were associated with COVID-19 anxiety. Women reported higher COVID-19 anxiety. All other variables were non-significant in the model. The model was statistically significant and explained 27% of the variance of COVID-19 anxiety: F (24, 858) = 12.56, p < 0.001, R2 = 0.27.

The final part of the analyses focused on changes over time explaining COVID-19 anxiety. The models are reported in Table 3. We found that increased psychological distress (β = 0.16, *p* < 0.001), increased technostress (β = 0.09, *p* = 0.003), and decreased social support from the work community (β = 0.09, *p* = 0.004) predicted higher COVID-19 anxiety. Finally, those who had changed their occupational area reported higher COVID-19 anxiety (β = 0.09, *p* = 0.006).

## 4. Discussion

This longitudinal research investigated the psychological, situational, and socio-demographic predictors of COVID-19 anxiety among Finnish workers. The results showed that perceived loneliness, psychological distress, technostress, and neuroticism were significant psychological predictors of COVID-19 anxiety of workers. It was also found that an increase in both psychological distress and technostress during the COVID-19 crisis predicted higher COVID-19 anxiety. Workers who had recently changed their field of work and expressed decreased social support from their work community reported higher COVID-19 anxiety. Of the demographic factors, female gender and younger age predicted higher coronavirus-related anxiety. These results support previous findings, indicating that those who feel lonely and individuals high in neuroticism, as well as females and younger people, are more vulnerable to experiencing COVID-19 anxiety [7,13,14,20].

According to earlier reports, Finnish workers’ mental health has not decreased dramatically due to the COVID-19 pandemic [33]. This could be an indication of the overall COVID-19 situation in Finland, which has been among the best in the world [65]. Finland is a very sustainable and stable society where workers’ rights and wellbeing are guaranteed, perhaps better than in some other countries [66]. However, significant predictors of COVID-19 anxiety among workers were identified in this study, as experiencing anxiety during stressful and uncertain times is normal [6]. Coping with the stressors and changes brought by the pandemic and managing and completing work tasks might result in increased anxiety with a continuity concern, as the state of emergency and restrictions are extended. Help and support from managers and colleagues as well as higher work engagement may protect workers from anxiety related to COVID-19 [33,45]. This might explain why, according to our study, those workers who had recently changed their field of work and those who expressed decreased social support had higher COVID-19 anxiety. It is conceivable that being in a new work environment amidst the pandemic can leave one feeling a lack of work engagement and support.

The literature further shows that the coronavirus pandemic and anxiety related to COVID-19 can disrupt workers’ mental wellbeing and hinder work performance [36,37]. This study found that higher COVID-19 anxiety and lower wellbeing at work during the pandemic are largely explained by psychological factors, including loneliness, psychological distress, technostress, and neuroticism. Naturally, the unknown and dangerous virus has evoked worries and anxiety among many, which has led to increased psychological distress. Moreover, COVID-19 has changed employees’ traditional working situations and challenged their social circumstances. This may have left them feeling alone and potentially isolated from their work community. Past research further indicates that neurotic individuals tend to respond more negatively to uncertainty and have poorer coping mechanisms in situations characterized by uncertainty [23,29]. This is consistent with our findings, as those higher in neuroticism were found to experience higher COVID-19-related anxiety.

Increased technology use has not entirely been able to maintain or create a meaningful psychological connection to work communities for those working remotely through the pandemic. In fact, increased use of technology has created additional stress and burden for many workers [48,49,50]. Maintaining an inclusive and caring work culture and providing technical and psychological support are crucial ways in which organizations can ensure the wellbeing of their employees in unprecedented and difficult times. We recognize that this may be challenging during times of crisis, however, the benefits for both the employees and the organization would be substantial. Previous research has underlined the impact social support received from work has on employees’ wellbeing [45,46] and identified that higher organizational support and social support are related to lower COVID-19 anxiety [35].

Such support can be delivered to workers through the use of multiple tools and methods. For instance, a study on Chinese workers indicated that having job autonomy in remote work during the pandemic can help prevent loneliness, which in turn prevents emotional exhaustion and declines in life satisfaction [67]. Autonomy may be needed for fostering online social interactions, which need to be organized and cannot happen as organically as they would in an offline workplace setting. Meanwhile, having one’s workday closely monitored can have a negative impact on employees’ wellbeing without enhancing their productivity. Therefore, in these unprecedented times, managerial practices may need to be reevaluated.

Maintaining psychological closeness to the workplace is also important for organizations. Pre-pandemic research suggests that it is the psychological, rather than physical, isolation that negatively impacts employees’ emotional connection with the workplace [68]. A lack of support and cohesion can be damaging in workplaces, particularly in populations that do not necessarily have similar regulated guarantees of wellbeing as Finnish workers. The areas of prevention and intervention are recognized, and support programs should be developed and provided to prevent and minimize collateral damage caused by COVID-19. Employers who foster communication via teleconferencing and other tools that mimic face-to-face communication in remote work may achieve benefits that help both their workers and the organization. Moreover, it was noted that employees’ personal preferences and personality should be taken into consideration, whenever possible, as highly disciplined individuals will have somewhat different needs than employees having trouble with self-discipline, or those low in emotional stability (e.g., high in neuroticism).

Our study provides additional insight in the predictors of COVID-19 anxiety among workers but is limited by using a single measurement of COVID-19 anxiety in one data collection period (autumn 2020). Future studies should investigate how such anxiety develops over a longer period of time. Out of the other variables included in the study, loneliness was also only measured once in the most recent data collected in autumn 2020. Hence, we could not analyze whether a change in loneliness had an impact on COVID-19 anxiety. In addition, our results are limited to the Finnish working population. Therefore, it is important to investigate COVID-19 anxiety in other countries using diverse population samples. The strengths of this study lie in its longitudinal design that enabled us to investigate changes from the pre-COVID-19 crisis era to the most recent developments. We had a high response rate, and the data are nationally representative of Finnish workers.

## 5. Conclusions

Drawing on a sample of Finnish workers, our results showed that COVID-19 anxiety was significantly associated with different psychological, situational, and socio-demographic factors. These results highlight the importance of recognizing that the COVID-19 pandemic gives rise to anxiety among workers and it may have serious implications for them. COVID-19 anxiety can negatively impact workers’ wellbeing, mental health, and work performance. Organizations should provide additional support to their employees and reassure them during a crisis.

## Figures and Tables

**Table 1:** Descriptive statistics of study variables.

	Range	M	SD	Ω
**Psychological**				
COVID-19 anxiety	6–42	18.91	6.76	0.87
Loneliness	0–6	1.76	1.65	0.85
Psychological distress	0–12	2.19	3.30	0.91
Technostress	6–42	12.89	7.41	0.92
Work exhaustion	0–30	14.26	7.57	0.92
Openness	3–21	14.71	3.32	0.70
Conscientiousness	5–21	15.63	3.04	0.70
Extroversion	3–21	13.52	4.32	0.87
Agreeableness	3–21	14.41	2.96	0.59
Neuroticism	3–21	11.69	3.64	0.74
**Situational**				
Social media information bubble	2–14	5.89	2.34	-
Social support from work	4–20	14.65	3.01	0.78
	*n*	%		
Remote work	391	37.45		
Lives alone	294	28.16		
**Socio-demographic**				
Female	472	45.21		
Age				
18–29	121	11.59		
30–49	518	49.62		
50–66	405	38.79		
High income	120	11.49		
University degree	496	47.51		
Occupational area				
Manufacturing	292	27.97		
Service	157	15.04		
Business, communic., and techn.	156	14.94		
Public administration	71	6.8		
Education	95	9.1		
Health and welfare	151	14.46		
Unknown	122	11.69		

**Table 2:** Predictors of COVID-19 anxiety among a national sample of Finnish workers.

	Models 0 (Age and Gender adj.)				Full Model			
	B	SE (B)	*p*	β	B	SE (B)	*p*	β
Psychological								
Loneliness	1.21	0.12	<0.001	0.30	0.47	0.16	0.005	0.11
Psychological distress	0.74	0.06	<0.001	0.36	0.36	0.08	<0.001	0.17
Technostress	0.242	0.028	0.000	0.27	0.15	0.04	<0.001	0.17
Work exhaustion	0.25	0.03	0.000	0.28	0.05	0.03	0.156	0.05
Openness	−0.02	0.06	0.706	−0.01	0.04	0.07	0.507	0.02
Conscientiousness	−0.21	0.07	0.003	−0.09	0.02	0.07	0.773	0.01
Extroversion	−0.19	0.05	0.000	−0.12	0.01	0.05	0.797	0.01
Agreeableness	−0.28	0.07	0.000	−0.12	−0.13	0.08	0.095	−0.06
Neuroticism	0.58	0.06	0.000	0.31	0.32	0.07	<0.001	0.17
Situational								
Social media information bubble	0.22	0.09	0.015	0.08	0.18	0.10	0.063	0.06
Social support from work	−0.40	0.07	0.000	−0.18	−0.10	0.07	0.184	−0.04
Remote work	0.87	0.43	0.043	0.06	0.01	0.46	0.980	0.00
Lives alone	−0.18	0.46	0.697	−0.01	−0.33	0.44	0.451	−0.02
Socio-demographic								
Female	2.11	0.42	0.000	0.16	0.91	0.45	0.044	0.07
Age (ref. 50–66-year old)								
18–29	1.95	0.69	0.005	0.09	0.64	0.69	0.352	0.03
30–49	0.86	0.44	0.053	0.06	0.16	0.46	0.732	0.01
High income	−1.43	0.65	0.029	−0.07	−0.39	0.64	0.537	−0.02
University degree	0.27	0.43	0.529	0.02	0.21	0.44	0.640	0.02
Occupational area (ref. manufact.)								
Service	0.88	0.67	0.189	0.05	0.10	0.66	0.877	0.01
Business, communic., and techn.	0.12	0.67	0.854	0.01	−0.01	0.59	0.986	0.00
Public administration	1.11	0.90	0.216	0.04	0.87	0.75	0.249	0.03
Education	0.18	0.80	0.821	0.01	−0.56	0.74	0.450	−0.03
Health and welfare	1.17	0.68	0.085	0.06	0.33	0.64	0.599	0.02
Unknown	0.79	0.73	0.278	0.04	0.51	1.52	0.735	0.01

Models 0 are adjusted only for age and gender.

**Table 3:** COVID-19 anxiety predicted by changes from pre-COVID-19 situation to current.

	% (yes)	B	SE (B)	*p*	β
Increased psychological distress	28.83	2.41	0.45	<0.001	0.16
Increased technostress	35.92	1.31	0.44	0.003	0.09
Increased work exhaustion	41.78	0.38	0.43	0.386	0.03
Increased information bubble	37.93	0.55	0.43	0.193	0.04
Decreased social support	39.48	1.27	0.44	0.004	0.09
Become remote worker	12.25	0.41	0.65	0.535	0.02
Became unemployed	3.35	1.05	1.14	0.360	0.03
Moved alone	1.92	1.28	1.51	0.395	0.03
Changed occupational area	19.79	1.48	0.53	0.006	0.09

All regression models are adjusted for age and gender. Change over time is measured using responses prior to (September–October 2019) and during the COVID-19 crisis situation (September–October 2020).

## Data Availability

The data presented in this study are available on request from the corresponding author. The data will be later on made publicly available at the Finnish Social Science Data Archive.

## Appendix A

The COVID-19 anxiety measure adapted from the STAI-6 [51] and designed to inquire about the respondents’ reactions to the situation caused by the coronavirus pandemic.

What kind of reactions does the coronavirus crisis evoke in you? Please evaluate, how well the next statements describe your state of mind during the past seven days.

I feel calm.

I am tense.

I feel upset.

I am relaxed.

I feel content.

I am worried.

Answer options are on a scale from 1 (does not describe my state at all) to 7 (describes my state completely). Statements 1, 4 and 5 were reverse coded for the analysis.
